# Associations between male reproductive characteristics and the outcome of assisted reproductive technology (ART)

**DOI:** 10.1042/BSR20170095

**Published:** 2017-06-27

**Authors:** Zhangshun Liu, Xiaohong Shi, Lihong Wang, Yan Yang, Qiang Fu, Minfang Tao

**Affiliations:** 1 Department of Reproductive Medicine Center, Shanghai Jiao Tong University Affiliated Sixth People’s Hospital, Shanghai 200233, China; 2 Department of Urology, Shanghai Jiao Tong University Affiliated Sixth People’s Hospital, Shanghai 200233, China

**Keywords:** Age, assisted reproductive technology, BMI, Male, Reproductive hormones, Semen parameters

## Abstract

The present study was designed to investigate the relationships between indicators of male body mass index (BMI), age, reproductive hormone levels, semen parameters, and the outcomes of assisted reproductive technology (ART). The clinical data were collected from 636 couples who underwent ART between January, 2013 and December, 2015 at the reproductive center involved in our study. Pearson’s correlation or Spearman rank correlation was applied to establish the relevant correlation coefficients. The correlation between influence factors’ and pregnancy outcomes was analyzed using the Logistic regression model. Analyses were conducted using SPSS software. Male BMI was found to be negatively correlated with testosterone (T) (*P*<0.05), while follicle-stimulating hormone (FSH) was negatively correlated with semen parameters (*P*<0.05). Luteinizing hormone (LH) was found to be negatively correlated with total sperm count, normal sperm morphology, and abortion (all *P*<0.05). Clinical pregnancy was related to sperm concentration and female age (*P*<0.05), and live birth was found to be associated only with female age (*P*<0.05). Male BMI was associated with the secretion of reproductive hormones, but had no effect on sperm parameters or ART outcome. A higher male age was also negatively connected with the outcome of clinical pregnancy. Reproductive hormones were not associated with ART outcome. Sperm concentration and female age were important factors influencing ART clinical pregnancy, while the only significant factor influencing live birth was female age. Levels of obesity-related inflammatory indicators (i.e. free fatty acid (FFA), glutathione peroxidase (GSH-Px), human inhibin-B (IHNB), interleukin-1 (IL-1), insulin-like growth factor-1 (IGF-1), and reactive oxygen species (ROS)) also varied with degrees of BMI. The present study provides information on the associations between male reproductive characteristics and the outcome of ART, which may contribute to improved strategies to help couples achieve better pregnancy outcomes.

## Introduction

Infertility is defined as the failure of conceiving after 12 months of unprotected intercourse, and it affects approximately 15% of couples trying to become pregnant [1,2]. Among couples between the age of 15–44 years old in the US, nearly 7.3 million women or their partners have sought infertility treatment services from 2006–2010 [3]. Although epigenetic and genetic causes are both determinants of successful embryo development, their effects on embryo quality from the male side have long been underestimated and insufficiently investigated [4,5]. According to former research, it is apparent that the rate of infertility rises with increased age in both males and females [6,7]. A previous case–cohort study reported the correlation between age and serum hormones or sperm parameters [8]. Recent evidence also suggest that many factors including obesity, physical activity, even snuff consumption, and education level seemed to have accumulative effects on sperm quality and the likelihood of pregnancy both spontaneously as well as after assisted reproductive technology (ART) [9]. Studies have shown that male lifestyle characteristics including social habits and diet, energy balance, hormonal deregulation, and spermatogenesis can all affect the sperm parameters and pregnancy of couples undergoing intracytoplasmic sperm injection [4,10–13].

Obesity, which is roughly defined as a body mass index (BMI) of more than 30 kg/m^2^, has been reported to be negatively associated with the reproductive capacity of both women and men [14,15]. Moreover, female obesity is found to passively affect fertility in regards to anovulation, risk of polycystic ovary syndrome, hormonal disturbances, and poor results after ART [16,17]. However, the relationships between male obesity and semen quality or outcome of ART are still uncertain. Some studies showed that increased male BMI passively affected live birth after *in vitro* fertilization treatments, while the correlation between BMI and live birth was less clear after intracytoplasmic sperm injection treatments [18,19]. Hammiche et al. [20] also showed that high male BMI could detrimentally influence sperm concentration and total mobile sperm count. It is suggested that obesity may influence male fertility by disturbing sperm DNA integrity [21], sperm quality [20], and by affecting hormones [22]. In contrast, Petersen et al. [19] found that there was little correlation between male BMI and sperm parameters, but that male BMI could affect pregnancy rate by influencing the quality of embryos. Hence, it remained dubious whether the loss of weight in males could improve assisted fertility success rates.

Besides this, reproductive hormone levels may have an important relationship with sperm quality and the chance of conceiving, yet the extent research evidence was also conflicting. Uhler et al. [23] showed that there was no significant relationship between reproductive hormones, including follicle-stimulating hormone (FSH), inhibin B, luteinizing hormone (LH), testosterone (T), and pregnancy or time taken to achieve pregnancy. Lahoud et al. [24] found that the addition of recombinant LH did not improve live birth or clinical pregnancy rates during IVF/ICSI. To the contrary, a meta-analysis showed that the addition of recombinant FSH may contribute to better successes in achieving pregnancy in women with low ovarian response. Meeker et al. [25] also suggested that FSH and LH were negatively correlated with sperm concentration, morphology, and motility, while inhibin B and free thyroxin (T4) were positively associated with sperm success parameters. Therefore, the mechanisms by which reproductive hormones such as T, FSH, LH, prolactin (PRL), and estrogen (E_2_) affect sperm quality and ART outcomes must be investigated to settle these existing uncertainties.

Finally, limited efforts have been made to elaborate the effects of possible risk factors on ART outcomes. Thus, we undertook the present study to explore the relationships between BMI, male age, reproductive hormones and sperm quality, clinical pregnancy, abortion, and live birth rate after intrauterine insemination (IUI).

## Materials and methods

### Subjects

The present study was conducted retrospectively at Shanghai Jiao Tong University Affiliated Sixth People’s Hospital between January, 2013 and December, 2015. A total of 636 couples were enrolled in the present study. All participants gave written informed consent. The serum reproductive hormone levels of 553 males were tested.

### Semen analysis

Every male participant provided three semen samples by masturbation, collected in a wide mouthed plastic container in a semen collection room at the reproductive center following a period of abstinence for 3–4 days. The semen samples were examined and processed according to the fifth edition of the World Health Organization (WHO) laboratory manual. Semen volume was calculated by aspiration into a graduated plastic beaker. Sperm concentration, total sperm count, progressive motility rate, and total sperm motility were measured using a computer-aided sperm analysis system (Beijing Weili New Century Technology Development Co., Ltd), and each sample contained more than 400 spermatozoa. The morphology of sperm was observed using modified Papannicolaou staining under a microscope (100×) with assessment of at least 200 spermatozoa in each sample. Semen analysis was performed by two specialized inspection technicians.

### Examination of reproductive hormones and inflammatory indicators

Fasted blood samples were withdrawn from the cubital vein of participants before 10:00 a.m. Any drug which could affect hormone levels was discontinued before blood sampling. After the separation of serum, the reproductive hormones (i.e. FSH, LH, T, PRL, and E_2_) were detected with chemiluminescence (Beckman Company, U.S.A.). Testosterone secretion indexes (i.e. TSI and T/LH) and testosterone–estradiol ratios (i.e. TE_2_ and T/E_2_) were calculated. Reproductive hormone examination was also performed by two specialized inspection technicians. The ELISA kits used in the measurement of free fatty acid (FFA), glutathione peroxidase (GSH-Px), human inhibin-B (IHNB), interleukin-1 (IL-1), insulin-like growth factor-1 (IGF-1), and reactive oxygen species (ROS) were purchased from the Xin’le Biology Ltd., Shanghai.

### BMI distribution

The height and weight of male participants were measured using an automated measuring instrument at the reproductive center. BMI was calculated according to the universal standard introduced by the World Health Organization (WHO). The 636 males included were divided into four groups: 15 in the underweight group (BMI < 18.5 kg/m^2^), 369 in the normal group (BMI 18.5–24.9 kg/m^2^), 210 in the overweight group (BMI 25–29.9 kg/m^2^), and 42 in the obese group (BMI > 30 kg/m^2^).

### Therapeutic method

IUI or IVF was used in all included couples. Human chorionic gonadotropin (HCG) was measured 2 weeks after the operation. An intrauterine sac observed by B-ultrasound at 4 weeks was defined as clinical pregnancy, and the parturition and fetal characteristics were also recorded.

### Post-operation follow-up

The cumulative clinical pregnancy rate = the number of pregnant couples/636, live birth rate = (the number of live births)/(the total number of live births and still births), and abortion rate = the number of abortion couples/636. The number of couples who achieved clinical pregnancy was 383, so the cumulative clinical pregnancy rate was 60.22%. The occurrence of spontaneous abortion was 66, so the abortion rate was 10.38%. A total of 334 couples successfully gave birth to 415 infants, so the cumulative birth rate was 52.52%.

### Statistical analysis

The Statistical Program for Social Science (SPSS 21.0) was used to complete statistical analyses. Quantitative data were normally distributed (Shapiro–Wilk test). Data in normal distribution were presented as mean ± standard deviation (mean ± SD), and those data not in normally distribution were expressed as median and quartile [Median (P_25_–P_75_)]. A* t*-test or nonparametric test was applied to determine the significance of differences. Frequency (constituent ratio) (F (%)) was used to describe categorical variables, and rank-sum test was used to characterize the significance of differences among groups. Applications of Pearson’s correlation or Spearman rank correlation were established to correlation coefficients. The associations of influence factors with pregnancy outcomes were analyzed using the logistic regression model. Differences were considered statistically significant when *P*-value was found to be <0.05.

## Results

### Baseline data analysis

The baseline data of the patients are given in Table 1.

**Table 1:** Baseline characteristics of the participants

Factors	Mean ± SD, median (P_25_–P_75_), or frequency (%)
Male age (years)	33.56 ± 5.72
22–29	160 (25.16%)
30–34	234 (36.79%)
35–39	145 (22.8%)
>40	97 (15.25%)
Infertility years	3.0 (2.0–5.0)
Smoking amount (/day)	
0	426 (66.98%)
1–10	127 (19.97%)
11–20	74 (11.64%)
21–40	9 (1.42%)
Height (cm)	172 (170–176)
Weight (kg)	70 (65–80)
BMI (kg/cm^2^)	22.2 (22.0–26.3)
<18.5	15 (2.36%)
18.5–24.9	369 (58.02%)
25–29.9	209 (32.86%)
>30	43 (6.76%)
Abstinence days	4 (3–5)
Sperm volume (ml)	3.6 (2.6–4.6)
Sperm concentration (10^6^/ml)	42.56 (24.93–71.12)
Total sperm amount (10^6^/ml)	153.18 (84.38–253.08)
Normal sperm morphology rate (%)	3.0 (2.3–4.1)
Progressive motility rate (%)	36.54 (24.02–48.12)
Total sperm motility (%)	43.19 (28.51–57.21)
FSH (IU/l)	4.38 (3.24–5.86)
LH (IU/l)	3.22 (2.36–4.51)
T (ng/ml)	3.35 (2.59–4.42)
PRL (ng/ml)	8.43 (6.19–11.51)
E_2_ (pg/ml)	31.29 (24.00–38.52)
TSL	1.07 (0.74–1.51)
TE_2_	0.11 (0.08–0.16)
Female age (year)	31.50 ± 4.68
2	81 (12.74%)

E_2_, estrogen; TE_2_, testosterone–estradiol ratio, T/E_2_; TSI, testosterone secretion index, T/LH.

### Association between BMI and baseline characteristics

It was observed that significant differences were found in between BMI and male age, T, TSI, and TE_2_ (*P*<0.05), yet no significant differences were identified in sperm volume, sperm concentration, total sperm amount, normal sperm morphology, total sperm motility, FSH, LH, PRL, and E_2_ (*P*>0.05, Table 2). It was shown that levels of obesity-related inflammatory indicators (i.e. FFA, GSH-Px, IHNB, IL-1, IGF-1, and ROS) were statistically quite distinct among patients classified in different BMI groups (*P*<0.05) (Table 3).

**Table 2:** Nonparametric tests for baseline characteristics based on BMI categories

Variable	Group				*Z*-test	*P*-value
	1 (*N*=15)	2 (*N*=369)	3 (*N*=210)	4 (*N*=42)		
Male age (years)	29 (25–32)	32 (29–36)	33 (30–37)	33 (28–36)	10.414	0.015
Infertility years	3 (2–5)	3 (2–5)	3 (2–4)	3 (2–5)	0.812	0.847
Sperm volume (ml)	4.2 (2.5–5.3)	3.7 (2.7–4.8)	3.4 (2.5–4.3)	3.2 (2.6–4.1)	6.711	0.082
Sperm concentration (106/ml)	40.42 (20.7–65.0)	42.54 (24.95–68)	42.76 (25.00–73.92)	45.65 (24.00–86.53)	0.981	0.806
Total sperm amount (106/ml)	104.86 (69.7–245.1)	161.57 (88.2–254.1)	153.18 (76.62–249.90)	121.8 (76.8–332.1)	1.434	0.697
Normal sperm morphology rate (%)	3.5 (2.4–4.4)	3.0 (2.1–4.0)	3.3 (2.4–4.4)	3.1 (2.4–4.0)	1.787	0.618
Progressive motility rate (%)	36.49 (24.61–54.65)	35.95 (23.35–47.85)	36.32 (23.66–49.71)	40.10 (26.97–46.58)	1.536	0.674
Total sperm motility (%)	38.40 (30.91–63.22)	42.49 (28.39–58.65)	44.16 (26.83–58.21)	47.94 (33.66–55.60)	1.249	0.741
FSH (IU/l)	3.92 (3.16–5.58)	4.33 (3.22–5.88)	4.47 (3.21–5.82)	4.57 (3.43–6.38)	1.000	0.801
LH (IU/l)	3.11 (2.07–3.72)	3.22 (2.46–4.60)	3.16 (2.2–4.37)	3.35 (2.43–4.56)	3.334	0.343
T (ng/ml)	4.87 (3.23–7.52)	3.78 (2.82–5.01)	2.90 (2.44–3.63)	2.61 (2.22–3.33)	64.823	0.000
PRL (ng/ml)	6.81 (5.61–16.29)	8.45 (6.14–11.59)	8.45 (6.36–11.33)	8.08 (6.00–11.72)	0.205	0.977
E_2_ (pg/ml)	35.12 (19.01–41.23)	30.99 (24.00–39.36)	30.99 (22.98–37.15)	35.34 (27.71–43.18)	5.500	0.139
TSI	1.66 (0.92–2.73)	1.14 (0.84–1.59)	0.98 (0.67–1.38)	0.76 (0.57–1.26)	24.371	0.000
TE_2_	0.13 (0.10–0.23)	0.13 (0.09–0.18)	0.10 (0.07–0.13)	0.08 (0.06–0.11)	42.794	0.000

E_2_, estrogen; TE_2_, testosterone–estradiol ratio, T/E_2_; TSI, testosterone secretion index, T/LH; BMI-based grouping was finally presented as: group 1 [median: 28; range (P_25_–P_75_): 26.25–29.00], group 2 [median: 32; range (P_25_–P_75_): 31–33], group 3 [median: 36; range (P_25_–P_75_): 35.0–37.5], and group 4 [median: 43; range (P_25_–P_75_): 41.0–45.5].

**Table 3:** Comparison of obesity-related inflammatory indicators of FFA, GSH-Px, IHNB, IL-1, IGF-1, and ROS within seminal plasma

Variable	Group				*P*-value
	1 (*N*=15)	2 (*N*=369)	3 (*N*=210)	4 (*N*=42)	
FFA (ng/ml)	306.05 ± 120.20	278.91 ± 95.78	428.69 ± 107.12	476.21 ± 117.35	<0.001
GSH-Px (U/ml)	101.85 ± 17.86	93.38 ± 24.05	76.45 ± 27.64	67.58 ± 23.85	<0.001
IHNB (ng/l)	28.24 ± 11.03	24.49 ± 8.94	38.47 ± 9.33	38.72 ± 11.16	<0.001
IL-1 (ng/l)	35.38 ± 10.57	39.44 ± 12.37	51.12 ± 11.20	51.26 ± 10.75	<0.001
IGF-1 (ng/ml)	86.58 ± 41.94	115.42 ± 49.16	145.92 ± 76.56	167.22 ± 55.90	<0.001
ROS (U/l)	350.40 ± 107.34	319.38 ± 156.31	567.58 ± 204.80	650.34 ± 194.64	<0.001

BMI-based grouping was finally presented as: group 1 [median: 28; range (P_25_–P_75_): 26.25–29.00], group 2 [median: 32; range (P_25_–P_75_): 31–33], group 3 [median: 36; range (P_25_–P_75_): 35.0–37.5], and group 4 [median: 43; range (P_25_–P_75_): 41.0–45.5].

**Figure 1:**
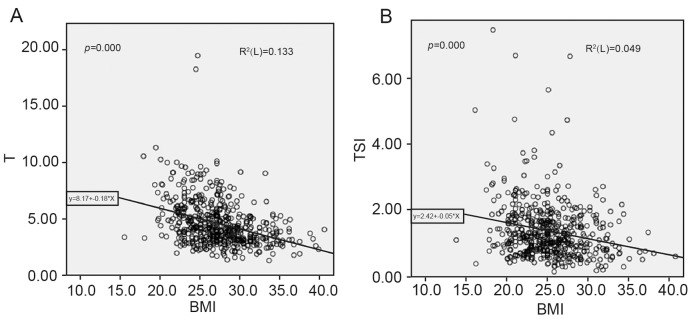
Linear regression analyses between T, TSI, and BMI (**A**) Relationship between BMI and T. (**B**) Relationship between BMI and TSI; TSI, testosterone secretion index from T/LH.

Furthermore, we found that BMI, T, and TSI were negatively correlated respectively (*P*<0.05). No statistically significant relationships were found between BMI and sperm parameters, other reproductive hormones, clinical pregnancy, abortion, or live birth rate (*P*>0.05) (Table 2). The correlations between T, TSI, and BMI were analyzed using the linear regression model, with T and TSI having a negative correlation with BMI (*P*<0.05) (Figure 1).

### Association between male age and baseline characteristics

Six-hundred and thirty-six participants were divided into four groups based on their BMI as follows: 15 people in group 1 (17.4–18.3), 369 in group 2 (21.2–24.3), 210 in group 3 (25.6–27.7), and 42 in group 4 (30.4–32.9). Accordingly, we found statistically significant differences in infertility duration, progressive motility rate, sperm volume, sperm motility, FSH, T, and PRL (*P*<0.05) (Table 4).

**Table 4:** Nonparametric tests for baseline characteristics based on male age categories

Variable	Group				*Z*-test	*P*-value
	1 (*N*=15)	2 (*N*=369)	3 (*N*=210)	4 (*N*=42)		
BMI (kg/cm^2^)	18 (17.4–18.3)	22.6 (21.2–24.3)	26.5 (25.6–27.7)	31.1 (30.4–32.9)	6.719	0.081
Infertility years	2.25 (2–3)	3 (2–4)	4 (2.00–7.25)	4 (2–6)	58.124	0.000
Sperm volume (ml)	3.75 (2.73–4.80)	3.60 (2.68–4.90)	3.60 (2.55–4.60)	3.2 (2.2–3.9)	11.423	0.010
Sperm concentration (106/ml)	38.62 (24.85–63.00)	41.55 (24.00–68.73)	49.00 (26.64–83.16)	42.76 (21.99–77.14)	4.947	0.176
Total sperm amount (106/ml)	148.41 (84.74–243.90)	153.18 (83.49–262.40)	167.70 (96.46–265.30)	148.80 (71.82–221.93)	4.429	0.219
Normal sperm morphology rate (%)	3.0 (2.3–4.3)	3.1 (2.4–4.0)	3.4 (2.1–4.2)	3.0 (2.0–4.0)	0.545	0.909
Progressive motility rate (%)	38.06 (24.81–48.21)	38.58 (26.93–50.00)	35.50 (23.96–47.30)	28.19 (17.36–45.13)	16.629	0.001
Total sperm motility (%)	43.52 (29.04–57.65)	46.15 (32.65–58.86)	41.74 (30.43–56.97)	33.53 (21.03–52.42)	14.154	0.003
FSH (IU/l)	4.21 (2.98–5.60)	4.02 (3.09–5.33)	4.41 (3.35–5.70)	5.86 (4.45–7.40)	37.393	0.000
LH (IU/l)	3.12 (2.29–4.34)	3.23 (2.29–4.52)	3.22 (2.37–4.41)	3.26 (2.46–4.66)	0.221	0.974
T (ng/ml)	3.42 (2.68–4.64)	3.34 (2.56–4.50)	3.45 (2.60–4.72)	3.03 (2.38–3.86)	8.24	0.041
PRL (ng/ml)	8.88 (6.45–12.30)	9.05 (6.47–11.79)	6.87 (5.49–9.09)	8.08 (6.47–12.29)	21.81	0.000
E_2_ (pg/ml)	32.77 (24.90–41.33)	31.10 (23.02–37.00)	32.55 (24.04–40.20)	29.07 (21.03–36.10)	6.543	0.088
TSI	1.14 (0.72–1.57)	1.08 (0.76–1.60)	1.11 (0.72–1.62)	0.98 (0.72–1.23)	5.664	0.129
TE_2_	0.11 (0.08–0.17)	0.11 (0.09–0.16)	0.11 (0.08–0.16)	0.11 (0.08–0.16)	1.979	0.577

TE_2_, testosterone–estradiol ratio, T/E_2_; TSI, testosterone secretion index, T/LH; Male age-based grouping was finally presented as: group 1 [median: 18.0; range (P_25_–P_75_): 17.4–18.3], group 2 [median: 22.6; range (P_25_–P_75_): 21.2–24.3], group 3 [median: 26.5; range (P_25_–P_75_): 25.6–27.7], and group 4 [median: 31.1; range (P_25_–P_75_): 30.4–32.9].

We also found that male age was associated with infertility duration, abstinence frequency, sperm concentration, total sperm motility, progressive motility rate, FSH, TSI, female age, clinical pregnancy, live birth rate, and the number of live births (*P*<0.05) (Table 5). Among all the associated significant factors, the progressive mobility rate, total mobility, TSI, clinical pregnancy, number of live birth rate, and number of live births were found to be negatively correlated with male age, while the other factors were correlated positively with male age (*P*<0.05).

**Table 5:** Correlation analysis between baseline characteristics and reproductive hormones

Variables	Statistics	FSH	LH	T	PRL	E_2_	TSI	TE_2_
Sperm volume	*r*	−0.028	−0.081	−0.008	0.020	−0.043	0.048	−0.067
	*P*	0.518	0.062	0.860	0.638	0.327	0.273	0.122
Sperm concentration	*r*	−0.199	−0.082	0.023	−0.060	0.075	0.056	−0.005
	*P*	0.000	0.058	0.598	0.168	0.083	0.194	0.910
Total sperm amount	*r*	−0.197	−0.144	0.000	−0.049	0.074	0.105	−0.044
	*P*	0.000	0.001	0.992	0.255	0.088	0.016	0.309
Sperm morphology rate	*r*	−0.110	−0.086	−0.036	0.028	−0.040	0.034	−0.020
	*P*	0.011	0.048	0.409	0.512	0.363	0.436	0.641
Progressive motility rate	*r*	−0.137	−0.076	−0.051	0.022	−0.021	0.001	−0.047
	*P*	0.002	0.081	0.237	0.614	0.632	0.981	0.283
Total sperm motility	*r*	−0.158	−0.084	−0.043	0.000	−0.013	0.012	−0.045
	*P*	0.000	0.053	0.319	0.996	0.762	0.775	0.295
Clinical pregnancy	*r*	−0.060	0.008	0.067	−0.065	0.025	0.073	0.039
	*P*	0.170	0.849	0.121	0.137	0.571	0.091	0.367
Abortion	*r*	−0.044	−0.292	−0.204	0.048	−0.033	0.185	−0.140
	*P*	0.750	0.029	0.131	0.727	0.808	0.172	0.304
Number of live birth	*r*	−0.064	0.022	0.048	−0.043	0.005	0.038	0.032
	*P*	0.141	0.608	0.271	0.941	0.913	0.385	0.467
Live birth	*r*	−0.078	0.043	0.086	−0.035	0.033	0.058	0.046
	*P*	0.070	0.326	0.048	0.414	0.447	0.182	0.285

E_2_, estrogen; TE_2_, testosterone–estradiol ratio, T/E_2_; TSI, testosterone secretion index, T/LH; * r*, correlation coefficient; *P, P*-value.

### Association between reproductive hormones and baseline characteristics

Statistically significant relationships were found between reproductive hormones and male age, BMI, sperm parameters, and ART outcome (Table 6). We found that FSH was negatively associated with sperm parameters, such as total sperm amount, sperm concentration, total sperm motility, progressive motility rate, and normal sperm morphology rate (*P*<0.05).

**Table 6:** Correlation analysis between baseline characteristics and clinical pregnancy

Variables	Pearson’s coefficient	*P-*value
Male age	−0.099	0.013
Infertility years	−0.073	0.064
Smoking amount	0.027	0.504
Abstinence days	−0.02	0.613
Sperm volume	−0.069	0.080
Sperm concentration	0.093	0.020
Total sperm amount	0.072	0.069
Normal sperm morphology rate	−0.001	0.975
Progressive motility rate	0.071	0.074
Total sperm motility	0.069	0.082
Female age	−0.127	0.001
Obesity	ref	ref

E_2_, estrogen; TE_2_, testosterone–estradiol ratio, T/E_2_; TSI, testosterone secretion index, T/LH.

**Figure 2:**
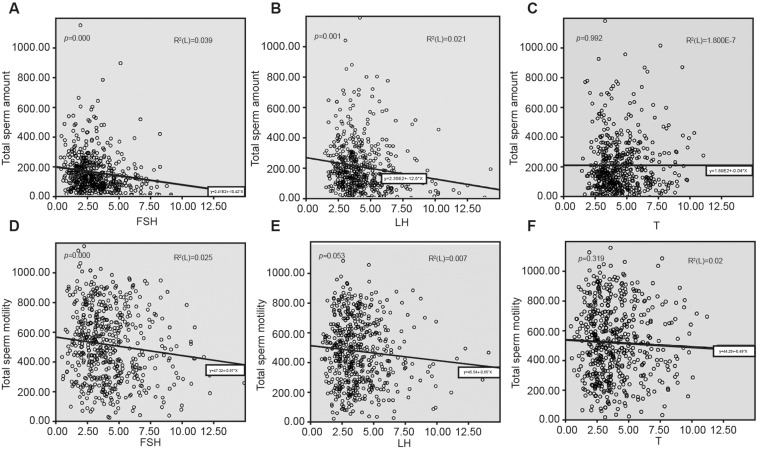
Linear regression analyses of total sperm count and motility with FSH, LH, and T (**A**) Association between total sperm count and FSH; (**B**) association between total sperm count and LH; (**C**) association between total sperm count and T; (**D**) association between total motility and FSH; (E) association between total motility and LH; (**F**) association between total motility and T.

In addition to this, LH was found to be negatively correlated with total sperm count, normal sperm morphology rate, and abortion (*P*<0.05); while T had no relationship with sperm parameters, but was positively related with the number of live births (*P*<0.05). Figure 2 shows the relationships between reproductive hormones (i.e. FSH, LH, and T) and sperm count or sperm motility.

### Association between clinical pregnancy success and baseline characteristics

When compared with clinical pregnancy outcome, statistically significant differences were found in male age, female age, and sperm concentration (*P*<0.05), while no statistically significant differences in the relations between infertility years, smoking history, abstinence frequency, sperm volume, total sperm count or other sperm parameters, and reproductive hormones were observed (*P*>0.05) (Table 6).

The logistic regression analysis of clinical pregnancy outcomes aimed at investigating the correlation between male age, sperm concentration, female age, and the clinical pregnancy outcome demonstrated that only female age and sperm concentration had effects on clinical pregnancy outcomes (Table 7).

**Table 7:** Logistic regression analysis of clinical pregnancy outcome

Factors	Unadjusted *P*	Adjusted *P*	OR
Male age	0.013	0.747	–
Sperm concentration	0.020	0.021	1.005
Female age	0.001	0.022	0.957

OR: odds ratio.

### Association between live births and baseline characteristics

To investigate the correlation between the relevant factors and live birth rates, we compared the live birth couples and the stillbirth couples, suggesting that higher male age, duration of infertility, and higher female age were all negatively correlated with live birth (*P*<0.05). No statistically significant association between smoking history, other sperm parameters, reproductive hormones, and live birth was observed (*P*>0.05) (Table 8).

**Table 8:** Correlation analysis between baseline characteristics and live birth

Variables	Pearson’s coefficient	*P-*value
Male age	−0.123	0.002
Infertility years	−0.093	0.018
Smoking amount	0.019	0.636
Abstinence days	−0.017	0.669
Sperm volume	−0.047	0.240
Sperm concentration	0.072	0.069
Total sperm amount	0.064	0.108
Normal sperm morphology rate	0.024	0.550
Progressive motility rate	0.078	0.049
Total sperm motility	0.074	0.061
Female age	−0.156	0.000
Obesity	ref	ref

E_2_, estrogen; TE_2_, testosterone–estradiol ratio, T/E_2_; TSI, testosterone secretion index, T/LH.

The logistic regression analysis was conducted to predict the correlation between the related factors and live birth, showing that only female age had substantial effects on live birth (Table 9).

**Table 9:** Logistic regression analysis of live birth

Factors	Unadjusted *P*	Adjusted *P*	OR
Male age	0.002	0.998	–
Infertility years	0.018	0.582	–
Progressive motility rate	0.049	0.232	–
T	0.048	0.058	–
Female age	0.000	0.002	0.941

T: testosterone.

## Discussion

At present, there are many conflicting evidence regarding the relationship between male BMI and sperm quality or ART outcome. Hence, the present study purposed to explore the respective associations of male age, BMI, and reproductive hormones with sperm parameters, clinical pregnancy, abortion, and live birth. After comprehensively analyzing the influencing factors and the outcome of pregnancy, we found that sperm quality and obesity-related inflammatory factors differed among groups with diverse levels of BMI, and only female age and sperm concentration were seen to affect the likelihood of pregnancy, as well as live birth.

It was shown that obesity-related inflammatory factors, namely FFA, GSH-Px, IHNB, IL-1, IGF-1, and ROS within seminal plasma, were discrepant in levels among groups with diverse levels of BMI, while no significant correlation was found between male BMI and sperm volume, concentration, amount, morphology, or motility [26,27]. In fact, IGF-1 has been found to elevate responses of the mesenchymal cells to HCG, through the increase in LH receptors on the surface of mesenchymal cells and the facilitation of additional cellular enzymes, finally affecting spermatogenesis and spermioteleosis [28]. GSH-Px was also instrumental in the initial stage of mitotic segregation of spermatogenesis, since it can limit the damage caused by ROS on sperm. It has also been demonstrated that IHNB was positively associated with the size of testes, and IHNB was actually an indicator of the spermatogenic ability [29]. Aside from this, cytokines such as IL-1, IL-6, and tumor necrosis factor (TNF) can lessen sperm motility and decrease the likelihood of sperm reaching the ovum, further contributing to infertility in males [30]. However, previous studies have tended to derive the conclusion that obesity is not associated with sperm quality, though one reported that men with BMI > 25 kg/m^2^ had fewer chromatin-intact normal-motile sperm cells per ejaculation [19,20,31]. Another study showed that a high male BMI could detrimentally influence sperm concentration and total mobile sperm count [20]. These studies simply summarized obesity as a BMI index value, but the use of BMI index to identify obesity is overly simplistic, as its related inflammatory factors could also be taken to represent obesity. Moreover, these studies did not involve other sperm quality parameters, such as semen volume, sperm morphology, sperm motility, and hormone levels. Furthermore, there was no significant change in sperm quantity but substantial decreases in mobility with increased age. In terms of hormones, FSH decreased with age, and male age was closely associated with sperm quality [32]. In contrast, Paasch et al. [8] demonstrated that higher age was correlated negatively with sperm parameters and serum hormones T and inhibin-B, but positively correlated with serum hormone FSH. These inconsistent results may be due to different treatment in our cohorts—for example, the infertility couples did not receive ART in their studies, but received ART in ours.

In this research, FSH had a negative correlation with sperm quality indicators such as total sperm count, sperm concentration, motility and morphology, and LH negatively affected total sperm count and morphology. Nonetheless, FSH, LH, Inhibin B, and T4 levels all were reported to be associated with sperm parameters including concentration, morphology, and motility [33–35]. This distinction may be attributed to the fact that their research was not based on hormone levels after ART, which implied the correlation between hormones level and infertility, but FSH, as a feasible treatment for infertility, may improve sperm DNA fragmentation and pregnancy success rate [36].

As for the critical factors affecting clinical pregnancy success and live birth, multiple regression analyses concluded that there was a negative association between higher female age and the chance of pregnancy, as well as live birth, while pregnancy could be seen to benefit from sperm concentration. Consistently, the cumulative live birth rate among older women was lower than that among younger women using vitrified blastocysts, suggesting that female age adversely influenced live birth rates [37]. It has also been strongly suggested that sperm concentration positively affects the likelihood of achieving pregnancy [38,39]. However, in the present study, we did not investigate whether or not female obesity could affect pregnancy and live birth after ART treatment, while another study showed that high female BMI indeed adversely affected live birth with the use of autologous oocytes, especially among women <35 years of age [17].

Our study assessed diverse factors influencing pregnancy and live birth. However, we have to acknowledge a few limitations in our study including its retrospective design and lack of uniformity between groups in the male participants who presented with potential causes of infertility. Therefore, we used multivariable analysis to control factors that covary with male BMI and clinical pregnancy for the purpose of minimizing latent unknown confounders. Besides, more indicators which could signify obesity other than solely BMI should be explored regarding their relationship with ABT outcomes so that potential predictors can be discovered and treatment methods more effectively managed. At last, the study of other female characteristics apart for age would almost certainly contribute to the comprehension of pregnancy outcomes of ART.

In summary, female age and sperm concentration were relevant factors affecting the likelihood of achieving pregnancy, and BMI is closely correlated with reproductive hormones but not sperm concentrations. Specifically, the correlation between reproductive characteristic and male BMI indicates that obesity will not impact ART outcomes significantly. The present study provides information on the associations between male reproductive characteristics and the outcome of ART, which may help couples achieve better pregnancy outcomes. However, further large ART-related cohort studies should still be conducted concentrating on the correlation between obesity and the likelihood of pregnancy and live birth.

## Informed Consent

Informed consent was obtained from all individual participants included in the present study.

## Abbreviations

ART: assisted reproductive technology
BMI: body mass index
FFA: free fatty acid
FSH: follicle-stimulating hormone
GSH-Px: glutathione peroxidase
HCG: human chorionic gonadotropin
IGF-1: insulin-like growth factor-1
IHNB: human inhibin-B
IL-1: interleukin-1
IUI: intrauterine insemination
LH: luteinizing hormone
PRL: prolactin
ROS: reactive oxygen species
TSI: testosterone secretion index, T/LH
